# Quality of diabetes care and health insurance coverage: a retrospective study in an outpatient academic public hospital in Switzerland

**DOI:** 10.1186/s12913-016-1801-z

**Published:** 2016-10-03

**Authors:** Yves Jackson, Juan Carlos Lozano Becerra, Marc Carpentier

**Affiliations:** 1 Division of primary care medicine, Department of community medicine, primary care and emergency medicine, Geneva University Hospitals, Geneva, Switzerland; 2 Global health institute, University of Geneva, Geneva, Switzerland; 3 Division of clinical epidemiology, Geneva University Hospitals, Geneva, Switzerland

**Keywords:** Diabetes, Quality of care, Health insurance, Coverage, Equity, Hospital

## Abstract

**Background:**

Socioeconomic disadvantage is associated with an increased risk of adverse diabetes outcomes. In Switzerland, a country with theoretical universal healthcare coverage, people without health insurance face barriers in accessing to and in receiving standard quality care. The Geneva University Hospitals (HUG) have implemented policies aiming at reducing these gaps. We compared quality of diabetes care and ambulatory healthcare services utilization among insured and uninsured diabetic patients.

**Methods:**

This retrospective study linked health and administrative data of type 2 diabetic outpatients with at least one HbA1c test performed in 2012–2013 at HUG. Quality of care evaluation relied on processes (annual serum HbA1c, cholesterol and microalbuminuria tesing) and outcomes (HbA1c) assessment. Healthcare utilization was assessed by the number of ambulatory clinical and laboratory visits. Results were stratified by disease course (newly diagnosed versus prevalent diabetes).

**Results:**

Of the 198 patients included, 80 (40.4 %) were uninsured. Both groups underwent annual testing of HbA1c, cholesterol, kidney function and microalbuminuria at comparably high rates and numbers of ambulatory visits did not significantly differ. After adjustments for age and sex, there were no significant differences in serum HbA1c between groups both in those with prevalent or with newly diagnosed diabetes. Initial medical intervention entailed comparable glycaemic improvement after 6 months in incident diabetes among insured and uninsured patients.

**Conclusions:**

This study did not find any difference in quality of diabetes care between insured and uninsured patients in a public hospital enforcing health-equity policies for access to and for delivery of standard diabetes care. It highlights the frontline role of public hospitals in contributing to care delivery equity even in countries with theoretical universal healthcare coverage.

## Background

Type 2 diabetes and its complications are a major cause of disability and of premature mortality worldwide, entailing a heavy economic burden for healthcare systems and society [1]. In developed countries, socioeconomic disadvantage is associated with a higher incidence of diabetes, of premature and severe complications of the disease and of avoidable hospitalizations and diabetes-related mortality [2, 3]. In Europe, diabetes-related death ratio is two times higher in immigrants than in local-born populations [4]. Mechanisms influencing this relationship are thought to relate both to individual factors, such as health behavior and access to care, and to the social and healthcare contexts in which individuals live [5]. Management of diabetes and prevention of complications predominantly rely on outpatients health services. Quality services positively influences diabetes outcomes, including incidence and progression of complications and mortality through preventive, educational and therapeutic interventions [6, 7]. In most countries, such benefits are not equally distributed among diabetics, reflecting disparities in access to those interventions and in the quality of services provided [8, 9].

Few evidences illustrate the relationship between health insurance coverage and diabetes outcomes, particularly in Europe. A major difficulty pertains to the close association between insurance coverage and other factors of socioeconomic vulnerability. In the United States, before the recent healthcare system reform, studies showed a gradient in quality of diabetes care and outcomes in groups with continuous, intermittent and absent coverage [8–10]. In the French universal healthcare system, despite more primary care services utilization, diabetics with low socioeconomic position get diagnosed at a later stage, suffer more complications and report being less empowered [11]. The 1994 healthcare law in Switzerland theoretically entails universal coverage with the enforcement of the legal obligation to purchase a private health insurance scheme for all persons in the country for more than 3 months. Yet, some groups, such as homeless, socially marginal people, undocumented immigrants or rejected asylum seekers, may not access to health insurance for economic or administrative reasons and therefore lack access to care [12]. To mitigate this gap, Geneva Canton implemented health equity policies aiming at facilitating access to high-quality care, including drugs, to all residents irrespective of insurance coverage, through a special scheme at the primary care division of the Geneva University Hospitals, the only public hospital in the Canton. Type 2 diabetes prevalence is rather low in Switzerland compared to neighboring countries [13]. Even when accessing to care and with insurance coverage, vulnerable immigrants are less likely to receive preventive measures for cardiovascular risk factors, including diabetes [14]. Moreover, insured diabetics from lower socio-economic status in Switzerland receive lower quality of care and have poorer outcomes, adding to similar inequalities found in patients with other chronic health conditions [13, 15–17].

This study aims at assessing quality of diabetes care in insured and in uninsured diabetic patients in an academic public outpatient facility in Geneva.

## Methods

### Setting

The Canton of Geneva in Western Switzerland had a population of 470,512 in 2012. An estimated 10,000 to 15,000 persons (undocumented immigrants, rejected asylum seekers, homeless) live in the Canton without health insurance [18]. The Geneva University Hospitals (HUG) are the largest healthcare institution in the canton. It provides full access to comprehensive primary care and specialized services for the whole population and is the port of entry into the healthcare system for vulnerable groups. Diabetic patients, irrespective of their insurance status, are usually attended by a multidisciplinary primary care team, under the supervision of specialists.

### Participants

The target population included all adult (≥18 years) patients with type 2 or gestational diabetes who received outpatient care at HUG from January 1st 2012 to December 31th 2013. Inclusion criteria included having had at least one serum HbA1c measurement during the study period and age below 65 (retirement age). Eligible participants were identified by systematically searching electronic medical records of all outpatients consulting during the study period for diabetes-related codes. Non-resident patients, such as temporary visitors or tourists, were excluded to compare only diabetics living in a similar geographical environment. Data linkage allowed crossing administrative and medical individual information.

### Design and instrument

This retrospective study explored the relationship between quality of diabetes care and socio-demographic (age, sex, origin) and administrative (insurance coverage) characteristics of outpatients seen at the public hospital. We evaluated quality of care both in terms of processes and of outcome. Processes under study included the number of visits to ambulatory care services (medical, nurse and laboratory), and annual testing for HbA1c, kidney function (serum creatinin and microalbuminuria) and serum cholesterol. The outcome measure was mean serum HbA1c levels. There was no plausibilisation or validation step before analyzing the selected data sets.

### Subgroup definition

For the outcome, we distinguished patients with prevalent diabetes at their first visit from those with newly diagnosed (incident) diabetes during the study period. A new diagnosis was considered in absence of mention of diabetes in the medical chart and of pathologic glycemic values prior to the study period.

### Definition of variables included in the analysis

European origin designates citizen of WHO Europe Region countries. New onset diabetes refers to disease not previously diagnosed or known. Follow-up is the interval between first and last HbA1c measure time points during the study period. Outpatient visit includes all clinical or laboratory encounters, irrespective of the medical reason. In the multivariate analysis, baseline time refers to the initial HbA1c measure time-point.

### Statistical analysis

We compared processes and outcomes of diabetes care between insured and uninsured. Descriptive statistics were computed for socio-demographic and healthcare utilization characteristics. Continuous variables are presented with mean and standard deviation and compared using Student’s *t*-test or Mann-Withney test in case of non normal distribution. Categorical variables are presented as percentage and compared with Chi-square test. Statistical significance was set at 5 %.

**Fig. 1:**
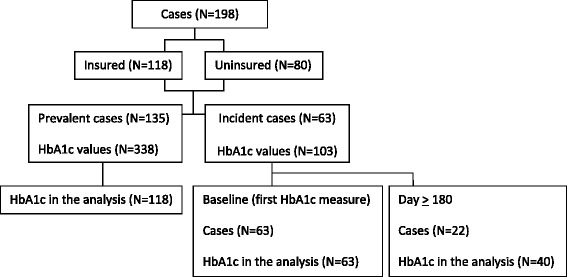
Cases and HbA1c measures included in the analysis

In order to better reflect the study population differences in terms of diabetes course and care, we conducted separated analysis regarding HbA1c (outcome) in those with newly diagnosed diabetes during the study period and those with a prevalent disease (Fig. 1). For prevalent diabetes, all HbA1c values were used. For newly diagnosed diabetes, only the baseline value and values after 6 months were modeled assuming a glycemic plateau would be reached after the initial therapeutic intervention. Multivariate mixed linear regressions were used to assess factors associated with diabetes control: a random effect was set for each patient; fixed effects were insurance status, gender, and age (less or more than 50 years); and for newly diagnosed patients a categorical fixed effect accounting for initial care, comparing values after 6 months to the baseline value Statistical significance level was set at 5 %. Statistical analyses were performed using CRAN R (version 3.2.0).

## Results

### Cases

A total of 198 diabetics were included in the study with 80 (40.4 %) being uninsured, 102 (51.5 %) women and with a mean age of 51.7 (standard deviation (SD): 8.2) years and originating from 57 different countries with European origin predominating. Overall, 135 (68.2 %) had prevalent diabetes at the beginning of the study period. Table 1 shows the main characteristics. Uninsured patients were more frequently female and of non European origin. They tended to be younger and to present more frequently with new onset diabetes. Mean follow-up duration was not significantly different between groups.

### Quality of diabetes care

#### Processes and ambulatory services utilization

Patients underwent 2.42 (SD: 1.49) HbA1c testing over the study period without any difference between groups (*p* = 0.481) (Table 2). Overall, 90.9, 85.6 and 83.3 % received annual HbA1c, serum cholesterol and kidney function testing, without significant differences between groups. Insured patients tended to have more outpatients visits than uninsured, although not significantly so.

### Outcome

#### Cases with newly diagnosed diabetes

In the multivariate analysis model, diabetes management during the first 6 months after diagnosis allowed for a mean HbA1c reduction of 2.10 % (95 % confidence interval: 1.30 to 2.90 %) (Table 3) without differences between insured and non insured patients (interaction between initial management and insurance effects: *p* = 0.2671). After 6 months, when we assumed stable HbA1c levels, insured patients had non-significantly (*p* = 0.5441) higher mean values (7.97 %; SD: 2.45 %) than uninsured ones (7.79 %; SD: 2.44 %). No association was found between HbA1c levels and age above 50 years (*p* = 0.8433) or sex (*p* = 0.1189).

#### Cases with prevalent diabetes

Of the 135 patients with prevalent diabetes, 89 (65.9 %) had two or more HbA1c tests. The median interval between the first and last available tests was 11 months (interquartile range: 6–17). While 39/89 (43.8 %) diabetics initially had HbA1c below 7 % indicative of good glycemic control, this proportion increased to 46/89 (51.7 %) (*p* = 0.307) at the time of the last test. In multivariate analysis, there was no significant association between mean HbA1c levels in the 135 prevalent cases and insurance status (*p* = 0.8459), sex (*p* = 0.9260) or age above 50 (*p* = 0.8846) (Table 4).

**Table 1:** Characteristics of the study population (*n* = 198) stratified by insurance status

	All cases N (%) or mean (SD)	Insured (*n* = 118) N (%) or mean (SD)	Uninsured (*n* = 80) N (%) or mean (SD)	*p-*value*
Age (year)	53 (11)	54.0 (9.8)	51.5 (10.0)	0.087 ^a^
Women	102 (51.5 %)	50 (42.4 %)	52 (65 %)	0.003
Origin (Europe)	64 (32.3 %)	54 (45.8 %)	10 (12.5 %)	<0.001
New onset diabetes	63 (31.8 %)	31 (26.3 %)	32 (40 %)	0.060
Follow-up (days)	116 (371)	123 (430)	114 (327)	0.684 ^b^

*SD* standard deviation

*Comparison between insured and uninsured groups

^a^ Student *t*-test7

^b^ Mann-Withney test

## Discussion

This study investigated the association between health insurance status and quality of diabetes care at an academic public outpatient facility in a country with theoretical universal healthcare coverage. It found no significant association, both in terms of processes and outcomes, among patients aged less than 66. Of note, more than 80 % of uninsured diabetics underwent the annual blood and urine tests recommended by international diabetes guidelines. This proportion was higher than previously reported from a consortium of public hospitals [19] and from population surveys conducted in the United States [8, 10]. Medical management during the initial 6 months following new diabetes diagnosis led to a sharp glycemic reduction irrespective of insurance coverage. This compares favorably with a representative sample of diabetics treated in the community in Switzerland showing only mild HbA1c improvement over a longer period [20]. It may reflect the “honeymoon” effect of early aggressive treatment. In our sample, no associations were found between HbA1c levels and insurance coverage, sex or age after 6 months of treatment. The factors under study showed no significant effect on steady state glycemic levels among prevalent cases either. Even though this absence of evidence is not the evidence of absence, this suggests a fair delivery of care irrespective of insurance coverage at the institution. Prevalent cases predominantly displayed mean HbA1c above the recommended target (HbA1c <7 %), without association with insurance and the proportion of well controlled participants did not differ significantly over the study period. A potential explanation for this stability pertains to the limited period of time along which our observations were conducted. Only half of the participants showed well controlled diabetes at their last HbA1c test. Previous hospital-based studies have not stratified glycemic control by disease course as we did making comparison with our data somewhat hazardous. For instance, Chew et al. showed that only 35 % diabetics of low socioeconomic status had well controlled diabetes in six US public hospitals [19]. Recent guidelines have underscored the limited clinical adequacy of too stringent HbA1c thresholds to assess diabetes control and have rather proposed objectives tailored to individual characteristics [7]. Finally, we found that insurance coverage had no influence over ambulatory health services utilization, highlighting the accessibility of care at the public hospital level and discarding concerns over possible abuse of enhanced accessibility for the most vulnerable patients.

**Table 2:** Diabetes quality of care and healthcare utilization in the study population (*n* = 198)

	All cases N (%) or mean (SD)	Insured (*n* = 118) N (%) or mean (SD)	Uninsured (*n* = 80) (%) or mean (SD)	*p-*value*
HbA1c tests (n)	2 (2)	2 (3)	2 (2)	0.4809
Annual HbA1c testing	180 (90.9 %)	105 (89 %)	75 (93.8 %)	0.3718
Annual cholesterol testing	170 (85.6 %)	102 (86.4 %)	68 (85 %)	0.9381
Annual kidney function testing	165 (83.3 %)	99 (83.9 %)	66 (82.5 %)	0.9484
Outpatient visits (n)	19.2 (18.1)	20.8 (21.6)	16.9 (10.9)	0.5330

*SD* standard deviation

*Comparison between insured and uninsured groups

While the Swiss healthcare system was designed to provide full coverage, real-life access to care is highly dependent on socioeconomic status, even among insured people. In Geneva, insured people of low socioeconomic position present more cardiovascular risk factors, while having more risk to forego care for financial reasons [21]. In other European or North American healthcare systems with universal access, this disadvantage is correlated with poorer glycemic control, increased risk of avoidable hospitalizations and lower quality of diabetes care [3, 11, 22]. Public hospitals may play a key equity role and efficiently complement private healthcare structures in delivering quality healthcare to the population in Western countries. Our study is the first to suggest an alleviation of disparities between insured and uninsured patients at a public hospital implementing specific health policies aiming at fostering equity in access to and delivery of standard diabetes quality of care, including access to antidiabetic agents. Further studies are necessary to assess the impact of such policies on other preventive and curative interventions. Before the healthcare reform in the US, some public hospitals implemented policies to enhance accessibility to and delivery of quality diabetes care for the most vulnerable groups including uninsured. While these programs afforded overall good results, disparities related to race and insurance coverage persisted, specifically for diabetes outcomes [19]. Similar inequalities remained in the provision of preventive diabetic care to vulnerable patients at federally qualified health centers with specific programs [23]. Of interest, those with partial coverage fared no better than the uninsured, highlighting the need of sustained access to care to attain optimal quality of care. Moreover, specific care programs and patients’ empowerment strategies can also positively impact on health disparities. In Germany, Baz et al. showed that diabetes control disparities between socioeconomic groups disappeared with structured managed care and health education in a cohort of patients attended at a tertiary-level hospital [24]. While our study was not designed to explore factors underlying this mitigating effect, several hypothesis can be drawn: a) the sustained political and economic support of health equity policies by local health and hospital authorities cascading down on to healthcare and administrative staff; b) the position of HUG as the only public Hospital within a large catchment area, allowing for streamlining clinical and administrative processes; and c) consistent, team-based and patient-centered care with registry-based information systems allowing for monitoring quality of care [25]. Our findings support the fact that public hospitals are at the forefront to mitigate diabetes-related health disparities in Western countries by fostering access to high quality care to vulnerable groups of patients.

**Table 3:** Multivariate analysis in cases (*n* = 63) with new onset diabetes

		N	HbA1 tests (n)	Mean HbA1c (SD)	*p*-value	Mean effect (95 % CI)
Time (days)	baseline	63	63	8.70 (2.72)	<0.001	0
	≥180	22	40	6.59 (0.98)		−2.10 (−2.90;−1.30)
Insurance	No	32	52	7.79 (2.44)	0.5441	0
	Yes	31	51	7.97 (2.45)		0.24 (−0.55; 1.03)
Sex	Men	26	42	8.20 (2.60)	0.1189	0
	Women	37	61	7.66 (2.31)		−0.64 (−1.46; 0.17)
Age (years)	<50	23	34	8.22 (2.75)	0.8433	0
	≥50	40	69	7.71 (2.27)		0.09 (−0.79; 0.97)

*SD* standard deviation

95 % CI: 95 % confidence interval

Access to health insurance remains problematic for some vulnerable groups of population even in countries with theoretical universal coverage. More than 100,000 persons lack health insurance in Switzerland with access to preventive care being highly variable between cantons [26, 27]. Enhanced access to insurance harnesses positive health and economics impact at population level, especially for people with chronic conditions by shortening delays in diagnosis and improving the management of complications [28]. Therefore, even though equity policy at hospital level may be beneficial in reducing inequalities, policy-making should ultimately aim at expanding insurance coverage universally nationwide to eliminate avoidable delays and complications.

**Table 4:** Multivariate analysis in cases (*n* = 135) with prevalent diabetes

		N	HbA1 tests (n)	Mean HbA1c (SD)	*p*-value	Mean effect (95 % CI)
Insurance	No	48	110	7.39 (1.92)	0.8459	0
	Yes	87	228	7.56 (1.68)		−0.06 (−0.67; 0.55)
Sex	Men	70	177	7.50 (1.66)	0.9260	0
	Women	65	161	7.51 (1.87)		−0.03 (−0.56; 0.61)
Age (years)	<50	46	103	7.51 (1.88)	0.8846	0
	≥50	89	235	7.50 (1.71)		0.05 (−0.57; 0.66)

*SD* standard deviation

95 % CI: 95 % confidence interval

Even though this study was not designed as an economic evaluation, our findings suggest the Geneva model may entail substantial benefits in terms of hospital and societal costs as compared to other setting without such facilitated access to public healthcare. Indeed, insured and uninsured patients had comparable ambulatory healthcare services utilization in Geneva. The lack of difference in HbA1c suggest comparable risks of diabetes-related complications, thus of the need of specialized and costly health interventions for underserved patients. In Switzerland, Constitutional rights guarantee access to emergency care in case of distress and uninsured patients account for a substantial proportion of emergency rooms users in public hospitals [29]. Those costs rest on institutions and cantons. Therefore, further studies are necessary to assess if ensuring access to standard care for uninsured people by may ultimately reduce societal expenses.

Several limitations should be considered. First, the number of uninsured diabetics is limited and may highlight the high proportion of undiagnosed diabetes in this population. Yet, we are fairly confident that the uninsured group was representative of the local context, as the HUG act as its only gateway to healthcare, we cannot exclude a selection bias among insured participants. Indeed, diabetic patients with insurance can seek care from private practitioners or private hospitals and disadvantaged patients often favor public hospitals. Second, the analysis was based on a limited set of variables failing to account for potential confounders in the relationship between insurance and glycemic control such as body-mass index, income, education, etc. This was due to the retrospective design of the study, the absence of diabetes quality of care monitoring system and of systematic socioeconomic data collection at HUG. Indeed, our data do not allow precisely assessing and discussing individual risk management. Moreover, the design entailed consequent drop-out rate in the incident cases follow-up, limiting the power of the multivariate analysis. Third, the lack of comparative data prior to the policy implementation hinders the assessment of a causal relationship between policies and the lack of disparities. Finally, this study was not built for non-inferiority objectives and the limited sample size might have incurred insufficient power and a risk of type 2 error obscuring potential differences between groups.

## Conclusions

In summary, this study did not show any difference in diabetes quality of care between insured and uninsured patients in a public hospital enforcing health-equity policies for access to and delivery of standard diabetes care. It highlights the frontline role of public hospitals in contributing to care delivery equity even in countries with theoretical universal healthcare coverage.

## Abbreviations

HbA1c: Glycated haemoglobin
HUG: Geneva University Hospitals
